# Nomogram prediction model for the clinical efficacy of flunarizine hydrochloride in patients with vertigo based on 5-HT and oxidative stress indicators^✰^

**DOI:** 10.1016/j.clinsp.2026.100936

**Published:** 2026-04-13

**Authors:** Hua Zhang, Xu Pan, Dan Lin, Xiaogang Yang, Puzhao Liu

**Affiliations:** Department of Otolaryngology, The First Affiliated Hospital of Henan University of Chinese Medicine, Zhengzhou, China

**Keywords:** Vertigo, Flunarizine hydrochloride, 5-hydroxytryptamine, Oxidative stress, Nomograms

## Abstract

**Objective:**

This study aimed to develop and validate a nomogram model integrating 5-Hydroxytryptamine (5-HT) and oxidative stress markers to predict the efficacy of flunarizine hydrochloride in treating vertigo.

**Methods:**

A retrospective study included 244 vertigo patients treated with flunarizine hydrochloride from September 2022 to December 2024. Patients were divided into training (70%) and validation (30%) sets. Clinical data, 5-HT levels, and oxidative stress indicators (Superoxide Dismutase [SOD], Malondialdehyde [MDA], Glutathione Peroxidase [GSH-Px]) were collected and analyzed. The influencing factors of treatment efficacy were screened using univariate and multivariate logistic regression. Least Absolute Shrinkage and Selection Operator (LASSO) regression was implemented using the R 'glmnet' package with 10-fold cross-validation. A nomogram model was constructed by integrating the independent influencing factors of treatment efficacy. Model performance was assessed via calibration curves, Consistency index (C-index), Decision Curve Analysis (DCA), and Receiver Operating Characteristic (ROC) curve.

**Results:**

There were 176 cases in the effective group and 68 cases in the ineffective group. Age, 5-HT, SOD, MDA, and GSH-Px were identified as independent predictors of treatment efficacy (*p* < 0.05). After variable selection via LASSO regression, the reduced nomogram demonstrated strong discriminative ability, with C-indexes of 0.862 (in the training set) and 0.874 (in the validation set), and average absolute errors of 0.144 and 0.138, respectively. Hosmer-Lemeshow tests confirmed good fit of the model (training: χ² = 9.061, *p* = 0.337; validation: χ² = 8.034, *p* = 0.430). ROC analysis yielded Area Under the Curve (AUC) values of 0.861 (95% CI: 0.797–0.925) and 0.870 (95% CI: 0.798–0.973) for training and validation sets, with sensitivity/specificity values of 0.892/0.711 and 1.000/0.650, respectively.

**Conclusion:**

The simplified nomogram incorporating age and oxidative stress markers demonstrates robust predictive accuracy while enhancing clinical feasibility. Actionable thresholds were established, such that patients with a score > 70-points should be considered for alternative therapies due to a high predicted risk of treatment non-response.

## Highlights

• First nomogram integrates 5-HT and oxidative stress markers for vertigo treatment.
• Model shows high accuracy (C-index 0.86‒0.87) and identifies key predictive factors.
• Facilitates personalized therapy by early detection of flunarizine poor responders.

## Introduction

Vertigo is a common clinical syndrome characterized by the illusion of movement in oneself or the surrounding environment, often accompanied by symptoms such as nausea and vomiting, which severely affects patients’ quality of life^1^ The etiology of vertigo is complex, involving the inner ear, cerebrovascular, cervical spine and other diseases. Flunarizine hydrochloride, a common drug for the treatment of vertigo, is a selective calcium channel antagonist that primarily inhibits L-type and T-type voltage-gated calcium channels in vascular smooth muscle and neuronal tissues. By doing so, it alleviates intracellular calcium overload and stabilizes cellular membranes. While its vasodilatory and vestibulo-suppressive effects are well-established, the interaction between calcium channel modulation and 5-HT pathways in vertigo remains unclear. Specifically, flunarizine dilates blood vessels to improve cerebral blood circulation and inhibits vestibular activity to enhance the blood circulation of vestibular organs^2^ However, the therapeutic efficacy of flunarizine varies considerably among individual patients. Studies suggest that 5-Hydroxytryptamine (5-HT) may modulate vertigo pathways, whereas flunarizine exerts its primary pharmacological effects through calcium channel blockade^3^ Preclinical studies have further demonstrated that flunarizine modulates 5-HT2A/2C receptors (Ki = 1.8 μM) and alters calcium channel sensitivity under oxidative stress, and findings that support the selection of 5-HT and oxidative stress markers (SOD, MDA, GSH-Px) as mechanisms of treatment response. As a key neurotransmitter, 5-HT is involved in the regulation of mood, cognition, and balance^4^ The fluctuation of 5-HT levels is not only related to migraine and depression conditions, frequently accompanied by dizziness symptoms, but also directly affects the function of the vestibular system, and abnormalities in this pathway will lead to balance disorders and dizziness^5^ Oxidative stress also plays a crucial role in the pathogenesis of vertigo, with Superoxide Dismutase (SOD), Malondialdehyde (MDA) and Glutathione Peroxidase (GSH-Px) recognized as core biomarkers of oxidative stress status. Our selection of these biomarkers is supported by existing evidence: 5-HT influences vestibular pathways via 5-HT2A/2C receptors, while oxidative stress markers (e.g., MDA) correlate with calcium channel dysfunction in animal models of vertigo. Alterations in these biomarkers can impair the function of nerve cells and vascular endothelial cells, and then affect the vertigo symptoms and the therapeutic effect of flunarizine hydrochloride^6^ A potential rationale for combining these biomarkers lies in synthesizing preclinical evidence: flunarizine has been reported to exhibit moderate affinity for 5-HT2A/2C receptors in radioligand binding assays, and oxidative stress is known to modulate calcium channel function^7^ Therefore, the authors hypothesized that 5-HT status and oxidative stress levels might influence or reflect the pathophysiological environment in which flunarizine exerts its calcium channel blockade effects, making them potential predictive candidates or treatment response. As an intuitive predictive tool, the nomogram quantitatively integrates multiple clinical variables to estimate the probability of a target clinical event. However, to date, no nomogram that combines 5-HT and oxidative stress indicators to predict flunarizine response in patients with vertigo has been developed. The present study addresses this critical knowledge gap by developing and validating a novel model, which aims to assist clinicians in implementing early individualized interventions, thereby potentially enhancing clinical outcomes and patients’ quality of life.

## Methods

### Study subjects

A total of 244 patients with vertigo who received flunarizine hydrochloride treatment in hospital between September 2022 to December 2024 were enrolled in this study. Inclusion criteria were defined as follows: 1) Meeting the diagnostic criteria of vertigo as specified in the “multidisciplinary expert consensus”^8^ ; 2) Aged from 18 to 80-years-old; 3) Provision of informed consent. Exclusion criteria included: 1) Presence of severe dysfunction of major organs (such as heart, liver, or kidney); 2) Suffering from a malignant tumor; 3) Administration of drugs that may affect 5-HT or oxidative stress levels within the preceding 3-months; 4) Mental comorbidity with disease or cognitive impairment that precluded completion of relevant inspection and assessment.

### Treatment protocol

All patients were administered flunarizine hydrochloride (manufacturer: xi 'an yeung sum pharmaceutical co., LTD., specifications: 5 mg/piece) treatment. The dosage was adjusted based on patient age: patients aged < 65-years received 10 mg once daily at bedtime, while those aged ≥ 65-years were given 5 mg once daily at bedtime. The treatment course was maintained continuously for 4-weeks.

### Observation indicators and detection methods

After 4-weeks of continuous treatment, the therapeutic effect was evaluated strictly in accordance with the criteria outlined in the 'Multidisciplinary Expert Consensus on the Diagnosis and Treatment of Vertigo and Dizziness'. Treatment outcomes were dichotomized into two categories: effective group (vertigo and associated symptoms were significantly ameliorated, with no restriction of daily living activities) or ineffective group (vertigo and associated symptoms showed no significant improvement or even aggravated). This dichotomous classification enhanced the standardization and objectivity of outcome assessment. Baseline general demographic and clinical characteristics of all patients were collected, including age, sex, Body Mass Index (BMI), smoking history (yes/no), alcohol (yes/no), complications, hypertension, diabetes, hyperlipidemia, yes/no). The serum levels of 5-HT, SOD, MDA and GSH-Px were detected by Enzyme-Linked Immunosorbent assay (ELISA). Standardized preanalytical protocols were implemented, including a unified 8-hour fasting status and fixed blood collection time (8:00‒10:00 AM), to minimize variability caused by dietary factors and diurnal rhythm. Peripheral 5-HT levels may not fully reflect central nervous system activity; thus, future studies are recommended to incorporate Cerebrospinal Fluid (CSF) or platelet 5-HT measurements. Additional oxidative stress markers (e.g., catalase, 8-OHdG) were not analyzed due to resource limitations. All ELISA kits were purchased from [Specific Biological Company] and operated in strict accordance with the manufacturer’s instructions. At the same time, routine blood tests (White Blood Cell Count [WBC], Red Blood Cell Count [RBC], Hemoglobin [Hb], Platelet count [PLT]) and blood biochemistry were detected Total Cholesterol (TC), Triglyceride (TG), Low Density Lipoprotein Cholesterol (LDL-C), High Density Lipoprotein Cholesterol (HDL-C), blood Glucose (GLU), Creatinine (Cr), Blood Urea Nitrogen (BUN) were performed for all enrolled patients.

### Methods of grouping

To mitigate selection bias, a consecutive series of patients who met the inclusion and exclusion criteria during the study period was enrolled retrospectively from the hospital's electronic database. Data were independently extracted by two researchers using a standardized case report form to ensure consistency and minimize information bias. Subsequently, the patients were divided into a training set (171 cases) and a validation set (73 cases) at a ratio of 7:3. The Nomogram predictive model was constructed in the training set, followed by internal validation of the model performance using the validation set.

### Statistical analysis

Data analysis was performed using IBM SPSS Statistics version 26.0. Continuous variables with normal distributions are presented as mean ± Standard Deviation (SD) and compared between groups using the independent samples *t*-test (for two-group comparisons). Categorical data are expressed as frequencies and percentages, compared with the Chi-Square test or the Kruskal-Wallis rank sum test for graded variables. Variables with statistical significance (*p* < 0.05) in univariate analyses were further included in a multivariate logistic regression model to identify independent factors associated with flunarizine treatment efficacy. The nomogram predictive model was developed using *R* software (version 4.2.2). Model performance was assessed through calibration curves, the concordance index (C-index), and Decision Curve Analysis (DCA). Actionable clinical thresholds were determined by maximizing Youden's index: a total score > 70 indicates high risk of treatment non-response, whereas a score < 30 indicates low risk, thereby providing clear and actionable guidance for individualized clinical decision-making. A two-tailed *p* < 0.05 was considered statistically significant.

To optimize variable selection, least absolute shrinkage and selection operator (LASSO) regression with 10-fold cross-validation was first applied to 12 candidate predictors, retaining 5 variables (λ = 0.021) with non-zero coefficients. Variable selection and modeling pipeline was implemented as follows: 1) Primary predictor screening: LASSO regression was implemented using the R 'glmnet' package (version 4.1.7) with 10-fold cross-validation. Minimum criteria (λ = 1 standard error of the minimum deviance) were applied to 12 candidate variables from univariate analysis. Five non-zero coefficients were retained: age (β = −0.079), 5-HT (β = 0.038), SOD (β = 0.029), MDA (β = −0.391), and GSH-Px (β = 0.026). To mitigate overfitting, the authors employed LASSO regression with 10-fold cross-validation (λ = 0.021) for predictor selection, retaining only age, 5-HT, SOD, MDA, and GSH-Px. Bootstrapping with 1000 resamples was performed to validate variable stability, showing selection frequencies > 80% for all retained predictors. 2) Variable stability validation: Bootstrapping with 1000 resamples was performed on the training set (*n* = 171). The selection frequencies for all retained variables exceeded 80%: age (92.3%), 5-HT (88.1%), SOD (85.7%), MDA (90.6%), GSH-Px (83.2%). 3) Final model construction: Multivariate logistic regression was conducted exclusively on LASSO-selected variables. The absence of multicollinearity among these variables was confirmed by tolerance values (> 0.1) and variance inflation factors (VIFs < 2.5). All predictors included in the final model exhibited significant associations with flunarizine treatment efficacy (*p* < 0.05 for all covariates). A nomogram was subsequently developed based on this validated multivariate logistic regression model.

While the number of ineffective events (*n* = 68) is below the conventional threshold for traditional logistic regression modeling, the integration of LASSO regularization with bootstrapping-based internal validation constitutes a recommended strategy for developing stable predictive models in such scenarios, as this approach explicitly penalizes model complexity and minimizes overfitting risk.

## Results

### Basic characteristics of patients in the training set and validation set

A total of 244 patients were enrolled in this study, among whom 176 (72.1%) were classified as treatment-effective and 68 (27.9%) as ineffective. All patients were randomly divided into a training set (*n* = 171, 123 effective and 48 ineffective) and a validation set (*n* = 73, 53 effective and 20 ineffective). As summarized in Table 1, the training and validation sets exhibited comparable baseline characteristics, with no statistically significant differences (*p* > 0.05) in demographic data, comorbidities, laboratory parameters, or biomarker levels.

**Table 1:** Comparison of baseline characteristics of patients in training set and validation set ($\bar{x}$±*s*).

Characteristics		Training set (*n* = 171)	Validation set (*n* = 73)	Statistical value	p
Age (years)		56.43 ± 10.24	55.87 ± 11.03	0.382	0.702
Gender	Male	92 (53.80)	39 (53.42)	0.002	0.957
	Female	79 (46.20)	34 (46.58)		
BMI (kg/m^2^)		24.36 ± 3.12	24.18 ± 3.34	0.404	0.636
Smoking history	Yes	47 (27.49)	19 (26.03)	0.055	0.814
	No	124 (72.51)	54 (73.97)		
Drinking history	Yes	38 (22.22)	16 (21.92)	0.002	0.957
	No	133 (77.78)	57 (78.08)		
Combined hypertension	Yes	60 (35.09)	22 (30.14)	0.562	0.453
	No	111 (64.91)	51 (69.86)		
Combined diabetes	Yes	37 (21.64)	14 (19.18)	0.187	0.665
	No	134 (78.36)	59 (80.82)		
Combined hyperlipidemia	Yes	48 (28.07)	21 (28.77)	0.012	0.911
	No	123 (71.93)	52 (71.23)		
WBC (× 10⁹/L)		6.87 ± 1.26	6.79±1.32	0.447	0.654
RBC (× 10¹²/L)		4.52 ± 0.48	4.48 ± 0.51	0.584	0.559
Hb (g/L)		130.25 ± 15.67	128.96 ± 16.34	0.581	0.561
PLT (× 10⁹/L)		220.36 ± 35.48	218.57 ± 37.21	0.355	0.722
TC (mmoL/L)		4.86 ± 0.87	4.82 ± 0.91	0.324	0.746
TG (mmoL/L)		1.87 ± 0.62	1.81 ± 0.64	0.685	0.493
LDL-C (mmoL/L)		2.89 ± 0.78	2.83 ± 0.82	0.541	0.588
HDL-C (mmoL/L)		1.24 ± 0.23	1.23 ± 0.27	0.294	0.768
GLU (mmoL/L)		5.67 ± 1.02	5.61 ± 1.08	0.413	0.679
Cr (μmoL/L)		78.36 ± 12.54	77.89 ± 13.02	0.265	0.791
BUN (mmoL/L)		5.23 ± 1.15	5.18 ± 1.21	0.306	0.759
5-HT (ng/mL)		110.52 ± 25.67	108.96 ± 27.34	0.426	0.670
SOD (U/mL)		90.36 ± 15.48	89.57 ± 16.21	0.359	0.719
MDA (nmoL/mL)		6.54 ± 1.87	6.48 ± 1.91	0.228	0.819
GSH-Px (U/mL)		115.67 ± 20.34	114.58 ± 21.02	0.379	0.704

### Univariate analysis of influencing factors of clinical efficacy of flunarizine hydrochloride in the treatment of vertigo patients in the training set

Univariate analysis of the training set revealed statistically significant differences in age, 5-HT, SOD, MDA, and GSH-Px levels between the treatment-effective and treatment-ineffective groups (*p* < 0.05; Table 2). These statistically significant variables were subsequently incorporated into a multivariate logistic regression model, with treatment efficacy (0 = effective, 1 = ineffective) as the dependent variable. Detailed variable coding assignments are provided in Table 3. The multivariate analysis identified age, 5-HT, SOD, MDA, and GSH-Px as independent predictors for treatment outcome (*p* < 0.05; Table 4). Collinearity diagnostics confirmed the absence of multicollinearity, with tolerance values > 0.1, Variance Inflation Factors (VIF) < 2.5, condition indices < 30, and no evidence of substantial variance proportion sharing (> 50%) among covariates. Furthermore, no significant interaction effects were observed between the included predictors, indicating that the influence of each factor on treatment efficacy was relatively independent.

**Table 2:** Flunarizine hydrochloride treatment of vertigo patients’ clinical curative effect of single factor analysis of influencing factors (plus or minus $\bar{x}$±*s*).

Characteristics		Ineffective (*n* = 48)	Effective (*n* = 123)	Statistical value	p
Age (years)		59.23 ± 10.12	54.17 ± 9.38	3.099	0.002
Gender	Male	26 (54.17)	66 (53.66)	0.003	0.952
	Female	22 (45.83)	57 (46.34)		
BMI (kg/m²)		24.43 ± 3.08	24.28 ± 3.25	0.275	0.783
Smoking history	Yes	12 (25.00)	35 (28.46)	0.206	0.649
	No	36 (75.00)	88 (71.54)		
History of alcohol consumption	Yes	11 (22.92)	27 (21.95)	0.018	0.891
	No	37 (77.08)	96 (78.05)		
Combined hypertension	Yes	21 (43.75)	39 (31.71)	2.198	0.138
	No	27 (56.25)	84 (68.29)		
Combined diabetes	Yes	15 (31.25)	22 (17.89)	3.636	0.056
	No	33 (68.75)	101 (82.11)		
Combined hyperlipidemia	Yes	12 (25.00)	36 (29.27)	0.311	0.576
	No	36 (75.00)	87 (70.73)		
WBC (× 10⁹/L)		6.89 ± 1.28	6.85±1.24	0.187	0.851
RBC (× 10¹²/L)		4.46 ± 0.49	4.53±0.48	0.851	0.395
Hb (g/L)		128.93 ± 16.02	130.68±15.24	0.665	0.506
PLT (× 10⁹/L)		218.87±36.18	220.72±35.23	0.306	0.759
TC (mmoL/L)		4.89±0.88	4.82±0.84	0.483	0.629
TG (mmoL/L)		1.89±0.63	1.85±0.61	0.381	0.703
LDL-C (mmoL/L)		2.91±0.79	2.87±0.76	0.305	0.760
HDL-C (mmoL/L)		1.22±0.27	1.26±0.21	1.029	0.304
GLU (mmoL/L)		5.72±1.05	5.64±1.03	0.453	0.650
Cr (μmoL/L)		78.77±12.78	78.13±12.42	0.300	0.764
BUN (mmoL/L)		5.26±1.16	5.21±1.13	0.258	0.796
5-HT (ng/mL)		94.67±20.33	114.83±22.17	5.465	0.001
SOD (U/mL)		84.73±14.28	92.37±15.13	3.013	0.003
MDA (nmoL/mL)		7.44±1.98	6.19±1.64	4.218	0.001
GSH-Px (U/mL)		107.76±18.32	120.31±21.28	3.597	0.001

**Table 3:** Variable assignment methods.

Variables	Meaning	Assignment
X1	Age	Continuous Variable
X2	5-HT	Continuous Variable
X3	SOD	Continuous Variable
X4	MDA	Continuous Variable
X5	GSH-Px	Continuous Variable
Y	Clinical efficacy of flunarizine hydrochloride in the treatment of vertigo	Effective = 0, Ineffective = 1

**Table 4:** Multivariate logistic regression analysis of influencing factors of clinical efficacy of flunarizine hydrochloride in the treatment of vertigo patients.

Indicators	β	Standard Errors	Wald	p	OR	95% CI
Age	−0.079	0.024	9.919	0.002	0.926	0.883‒0.972
5-HT	0.038	0.011	11.539	0.001	1.039	1.016‒1.062
SOD	0.029	0.014	4.281	0.039	1.030	1.002‒1.059
MDA	−0.391	0.124	9.990	0.002	0.676	0.530‒0.862
GSH-Px	0.026	0.012	4.848	0.028	1.026	1.003‒1.050

Note: Variables retained after LASSO regression with selection frequencies: age (92.3%), 5-HT (88.1%), SOD (85.7%), MDA (90.6%), GSH-Px (83.2%).

### Construct a nomogram prediction model

**Fig. 1:**
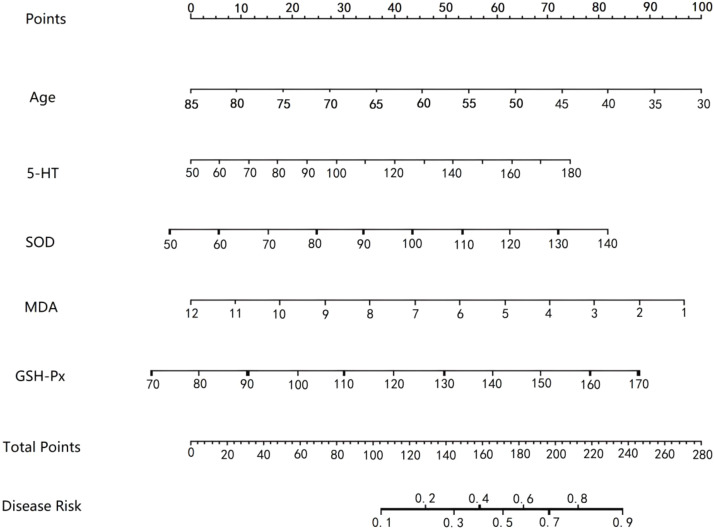
Nomogram prediction model based on 5-HT and oxidative stress indicators.

Based on the independent predictors identified by multivariate logistic regression analysis, a Nomogram prediction model was constructed to predict the clinical efficacy of flunarizine hydrochloride in the treatment of patients with vertigo. Internal validation via bootstrapping confirmed the robustness of the model, with predictor selection frequencies as follows: age (92.3%), 5-HT (88.1%), SOD (85.7%), MDA (90.6%), and GSH-Px (83.2%). A scoring scale was assigned to each predictor based on its regression coefficient, and the total score of the nomogram corresponds to the predicted probability of ineffective treatment of patients (Fig. 1).

### Evaluation and validation of nomogram prediction model

**Fig. 2:**
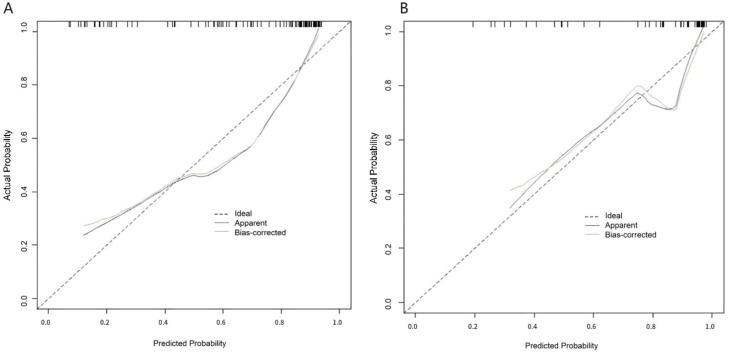
Calibration curve in the training set (A) and the verification set (B).

**Fig. 3:**
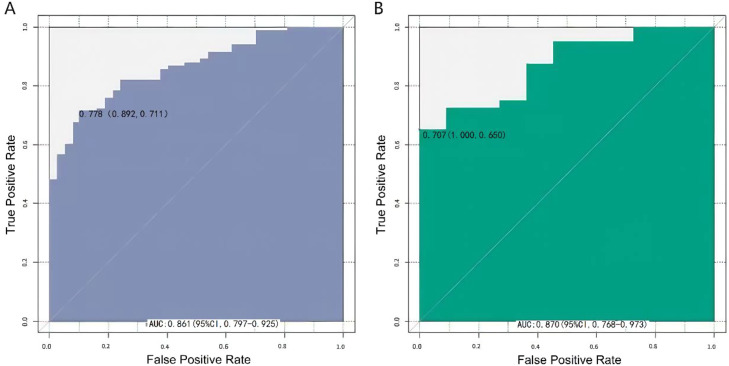
ROC curve in the training set (A) and the verification set (B). Note: (A) The AUC is 0.861, with a 95% CI of 0.797–0.925. (B) The AUC is 0.870, with a 95% CI of 0.768–0.973.

In the training set and validation set, the C-index of the Nomogram prediction model was 0.862 and 0.874, respectively. Further calibration curve analysis showed that the predicted values of the Nomogram model were in good agreement with the actual observed values, as evidenced by the mean absolute error of 0.144 and 0.138, respectively (Fig. 2). The model calibration curve is relatively close to the ideal line, indicating good consistency between the model's predicted probabilities and the actual results. Furthermore, the results of the Hosmer-Lemeshow test showed that the χ^2^ values of the training set and the validation set were 9.061 (*p* = 0.337) and 8.034 (*p* = 0.430), respectively. The predictive performance of the Nomogram was further evaluated using ROC analysis. The AUC values of the training set and validation set were 0.861 (95% CI: 0.797‒0.925) and 0.870 (95% CI: 0.768‒0.973), respectively (Fig. 3). The corresponding sensitivity and specificity combinations were 0.892, 0.711 and 1.000, 0.650, respectively. Clinical actionable thresholds were determined by maximizing Youden's index: in the training set, a total nomogram score > 70 was defined as high risk of treatment non-response (specificity = 89%), and a score < 30 was defined as low risk (sensitivity = 92%). These results showed that the model not only exhibited good performance on the training set, but also maintained good generalization ability on the validation set. While the robust performance in internal validation is encouraging, it should be acknowledged that the relatively small sample size of the validation set (*n* = 73) limits the statistical power for robust generalization of the model’s efficacy.

### Decision curve analysis of nomogram prediction model

**Fig. 4:**
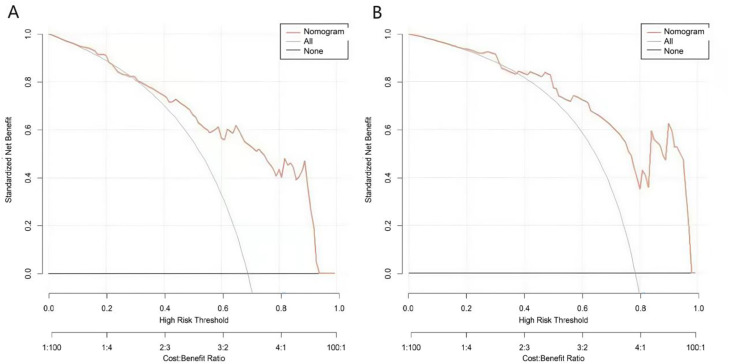
Decision curve in the training set (A) and the verification set (B). Note: The “Nomogram” curve in the figure represents the net benefit of the nomogram model; the “All None” curve represents the net benefit of the extreme strategy (i.e., intervening in all cases or not intervening in any case).

The decision curve revealed that the nomogram provided a net benefit across a wide threshold probability range of 0.05 to 0.93 (Fig. 4), indicating its potential clinical utility. To translate these findings into actionable guidance, the authors refer to the nomogram (Fig. 1). Based on the distribution of total nomogram scores and the corresponding predicted probabilities of treatment non-response, the authors propose a clinically actionable scoring threshold: a patient's total nomogram score of > 70-points correspond to a high predicted probability of treatment non-response (> 70%). For such patients, clinicians should strongly consider alternative or adjunctive therapies rather than proceeding with flunarizine monotherapy. Conversely, a total score < 30-points indicate a high likelihood of treatment response, which supports the initiation of flunarizine therapy.

### Exploratory subgroup analysis by etiology

Given the potential heterogeneity in vertigo etiologies, the authors conducted a post-hoc exploratory analysis on the two largest subgroups with clear diagnostic documentation: Vestibular Migraine (VM, *n* = 58) and Meniere's Disease (MD, *n* = 41). While the small sample sizes preclude definitive conclusions, the direction of association for the predictors (Age, 5-HT, SOD, MDA, GSH-Px) was consistent with those in the main model across both subgroups. However, the magnitude of the odds ratios varied, suggesting that vertigo etiology may act as an effect modifier. This potential effect modification warrants further investigation in larger, dedicated etiological subgroup studies.

## Discussion

Vertigo, a common clinical syndrome, severely impairs the quality of life of patients^9^ Flunarizine hydrochloride is a widely used drug for the treatment of vertigo; however, its therapeutic effect varies considerably among individual patients^10^ Our study identifies serum levels of 5-HT and oxidative stress markers as independent predictors of treatment response to flunarizine. Although the precise mechanistic interplay between these biological systems and flunarizine’s primary calcium channel-blocking action remains to be fully elucidated, several plausible connections can be considered^11^^,^^12^ This study aimed to develop and validate a nomogram incorporating 5-HT and oxidative stress indicators to guide personalized therapeutic strategies for vertigo.

In this study, 244 patients with vertigo treated with flunarizine hydrochloride were included, including 176 cases of effective treatment and 68 cases of ineffective treatment. Multivariate logistic regression analysis revealed that age, 5-HT, SOD, MDA and GSH-Px were independent predictors for the clinical efficacy of flunarizine hydrochloride in the treatment of vertigo patients (*p* < 0.05). As an important factor, age was found to correlate with treatment response: with the increase of age, the physiological functions of the human body gradually decline, including nerve cell function, vascular elasticity, and so on^13^ This may alter the body’s metabolism of and response to flunarizine hydrochloride, thereby influencing the therapeutic effect^14^ For instance, the blood-brain barrier function may be weakened in the elderly, which may change the amount and rate of drug entry into the brain and affect the improvement of vertigo symptoms^15^

As a key neurotransmitter, 5-HT exerts a direct regulatory effect on the vestibular system. When the level of serotonin is abnormal, it may cause imbalance and dizziness^16^ In our study, 5-HT levels were significantly associated with therapeutic efficacy. This suggests that serotonergic fluctuations may modulate flunarizine's activity in vestibular pathways or its metabolic processing, thereby regulating the drug's clinical effectiveness.

The changes of oxidative stress indicators such as SOD, MDA and GSH-Px reflected the state of REDOX balance in the body^17^ SOD is an important antioxidant enzyme that scavenges superoxide anion free radicals in the body and protects cells against oxidative damage^18^ MDA is a product of lipid peroxidation, and its elevated levels indicate increased oxidative stress in the body^19^ GSH-Px is involved in the maintenance of REDOX balance in cells^20^ In patients with vertigo, oxidative stress imbalance may impair the function of nerve cells and vascular endothelial cells, thereby interfering with the therapeutic efficacy of flunarizine hydrochloride^21^ One hypothetical explanation for the association between oxidative stress and treatment response is that an enhanced oxidative stress state may induce vascular endothelial dysfunction. This, in turn, could impair cerebral blood flow and consequently reduce drug delivery to the target site.^22^

Notably, the mediating role of oxidative stress in linking underlying pathologies to clinical outcomes is not unique to vertigo. Recent studies have confirmed that oxidative stress serves as a key intermediate in thyroid pathology and gestational diabetes; its overactivation triggers tissue damage and ultimately leads to preterm birth,^23^ highlighting the conserved pathological significance of oxidative stress imbalance across diverse disease entities. Mechanistically, excessive oxidative stress has been shown to disrupt potassium-ATP channel function, which is closely involved in regulating cellular energy metabolism and resisting reperfusion injury^24^ This finding implies that the therapeutic response to flunarizine in vertigo patients may partially depend on the drug’s potential to modulate oxidative stress-related ion channel signaling. Furthermore, ROS, the core effector molecules of oxidative stress, play an indispensable role in the mechanisms of ischemic preconditioning and postconditioning,^25^ which are critical adaptive responses in cerebral ischemia-a common pathological basis of vertigo. Collectively, these updated evidences reinforce our hypothesis that oxidative stress-induced vascular endothelial dysfunction and impaired cerebral blood flow are key factors affecting the therapeutic efficacy of flunarizine hydrochloride.

The Nomogram demonstrated good discriminatory ability, with C-index values of 0.862 in the training set and 0.874 in the validation set. Calibration curves analysis further indicated good agreement between predicted probabilities and observed outcomes, supported by low mean absolute errors (training set: 0.144; validation set: 0.138), which reflect the model's high reliability. The Hosmer-Lemeshow goodness-of-fit test revealed χ^2^ values of 9.061 (*p* = 0.337) for the training set and 8.034 (*p* = 0.430) for the validation set; all p-values were > 0.05, indicating good fit of the model to the actual data. The predictive performance of the Nomogram was further evaluated using ROC analysis. The AUC in the training set and validation set were 0.861 (95% CI: 0.797‒0.925) and 0.870 (95% CI: 0.768‒0.973), respectively, indicating the model’s favorable prediction performance. Corresponding sensitivity and specificity combinations of the training set and the validation set were 0.892, 0.711 and 1.000, 0.650, respectively. Given the limitations of retrospective designs, future prospective studies with stratified randomization by vertigo etiology (e.g., vestibular vs. cerebrovascular) are needed to mitigate selection bias. Post-hoc power analysis indicated that a minimum requirement of 150 samples for the validation set would be required to achieve 80% statistical power. Additionally, the number of non-responsive events (*n* = 68) was relatively small compared to the 5 included predictors ‒ despite the use of LASSO regression and bootstrapping to reduce overfitting risk, this imbalance may still introduce potential overfitting. Thus, the current validation set (*n* = 73) may lack sufficient power to draw robust conclusions. Although the model exhibited robust discriminatory ability, its reliance on non-routine biomarkers (e.g., 5-HT and oxidative stress assays) may hinder widespread clinical implementation. Future studies should prioritize the integration of routinely accessible clinical parameters, such as vertigo severity scales and comorbidities, and include comparative efficacy analyses of flunarizine versus first-line therapeutic agents (e.g., betahistine).

The model's performance across various sensitivity-specificity thresholds enables clinicians to select an optimal cut-off value based on specific clinical needs. This flexibility enhances its utility in personalizing patient care. For instance, patients identified as high-risk for poor treatment response could be preemptively managed with alternative agents, dose adjustments, or combination therapies. However, the clinical implementation of the current nomogram is presently limited by its reliance on biomarkers (5-HT, SOD, MDA, GSH-Px) that are not routinely measured in standard vertigo management. Therefore, our model should be interpreted as a proof-of-concept that validates the predictive value of serotonergic and oxidative stress pathways in flunarizine response. For future clinical translation, research should focus on two avenues: 1) Validating these findings in larger, independent cohorts to justify the potential cost and effort associated with biomarker testing in specific patient subgroups; and 2) Exploring whether these biological insights can be approximated or substituted with more readily available clinical or laboratory parameters.

This study developed and validated a nomogram that incorporates 5-HT and oxidative stress indicators to predict the therapeutic efficacy of flunarizine hydrochloride in patients with vertigo. The model demonstrates robust discriminatory power, providing a valuable tool for the early identification of patients at high risk of poor treatment response and for guiding personalized treatment strategies. While our data support a predictive role for 5-HT, it is important to contextualize this finding within conflicting evidence. For instance, a comparative study by Zhu et al. (2023) focused on migraine relief and reported that another agent (ratanasampil) was superior to flunarizine in efficacy, without specifically identifying baseline 5-HT levels as a predictor of flunarizine response^26^ This discrepancy may be attributed to fundamental differences in study design and primary focus. Notably, Zhu et al.'s study was a comparative efficacy trial centered on migraine, which likely involved a distinct patient phenotype. In contrast, our study specifically aimed to develop a predictive model for flunarizine response in a broader vertigo cohort by integrating biomarker profiles. It is plausible that the role of the serotonergic system in modulating vestibular signal processing and drug response is more pronounced in certain vertigo subtypes or under specific pathophysiological conditions. This suggests that the predictive power of 5-HT might not be uniform across all study populations or clinical endpoints. Our finding, therefore, may be most applicable to a broader, less pre-selected patient population presenting with vertigo and highlights the need for etiology-stratified and biomarker-informed analyses in future research.

This study has several limitations: Firstly, the mechanistic link between 5-HT pathways and flunarizine's calcium channel blockade remains speculative, requiring in vitro/in vivo validation of potential receptor interactions. Secondly, while oxidative stress markers (SOD, MDA, GSH-Px) were associated with treatment outcomes, these markers are nonspecific and can be influenced by numerous confounders (e.g., diet, inflammation, comorbidities). Their changes may be epiphenomenal rather than causative, highlighting the need for longitudinal studies with temporal biomarker measurements. Thirdly, despite efforts to enroll consecutive patients and adopt standardized data extraction protocols, the retrospective study design still carries a risk of residual selection and information bias (e.g., from unmeasured confounders such as detailed medication adherence or specific lifestyle factors). This precludes causal inferences and necessitates validation in a prospective setting. Fourthly, this is a single-center, retrospective study; thus, the model requires external validation in multi-center, prospective cohorts with standardized biomarker protocols to confirm its generalizability and calibrate it for diverse patient populations and clinical settings. This is a crucial prerequisite for any future clinical application. Additionally, the study lacked comparative efficacy analysis with other first-line vertigo treatments (e.g., betahistine), which limits the clinical reference value of the model. Future studies should incorporate such comparative data. Fifthly, the binary outcome measure, though based on clinical consensus, is inherently subjective and lacks the precision of a validated vertigo-specific scale (e.g., DHI). This may introduce misclassification bias and reduce the granularity of our efficacy assessment. Future prospective studies should incorporate such validated scales to define treatment response more objectively and reliably. Furthermore, while the etiological heterogeneity of our vertigo cohort enhances the generalizability of the model to an undifferentiated patient population in a general clinic, it may also dilute stronger biomarker associations present in specific vertigo subtypes. Our exploratory subgroup analysis suggests that the predictive strength of the model's components might vary across different etiologies. Therefore, external validation and refinement of this model in well-defined, homogeneous patient cohorts (e.g., pure Vestibular Migraine cohorts) are the next crucial steps to enhance its clinical precision and applicability.

## Conclusion

The simplified nomogram incorporating age and oxidative stress markers demonstrates robust predictive accuracy while enhancing clinical feasibility. Clinically actionable thresholds were established: patients with a total score > 70-points should be considered for alternative therapies due to high predicted non-response risk of treatment non-response; conversely, those < 30-points are highly likely to benefit from flunarizine.

## Ethics approval and consent to participate

The study was approved by the Committees for the Ethical Review of Research involving Human Subjects from First Affiliated Hospital of Henan University of Traditional Chinese Medicine (Approval n°HNTCM-20,222,454). Written informed consent specifically included permission for biomarker testing (5-HT, SOD, MDA, GSH-Px) using serum samples, with detailed explanations of laboratory procedures and data anonymization. All procedures adhered to the Declaration of Helsinki.

## Clinical trial number

Not applicable.

## Authors’ contributions

Conception and design: HZ and XP. Method: DL. Data Collection: HZ. Manuscript Writing: HZ and XGY. Manuscript revision: HZ, XP and PZL. Research supervision: HZ and XP. All authors contributed to the article and approved the submitted version.

## Declaration of competing interest

The authors declare no conflicts of interest.
